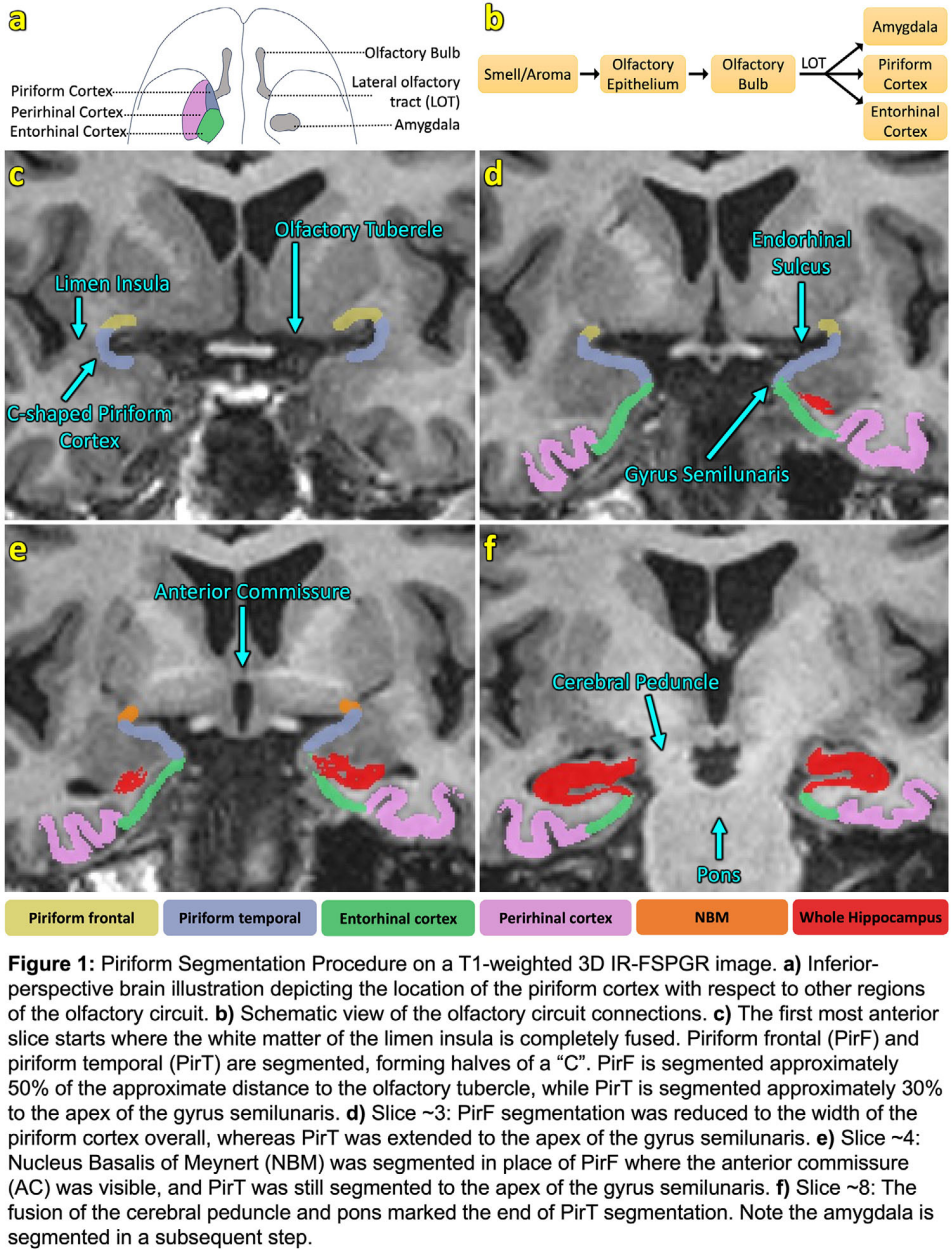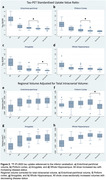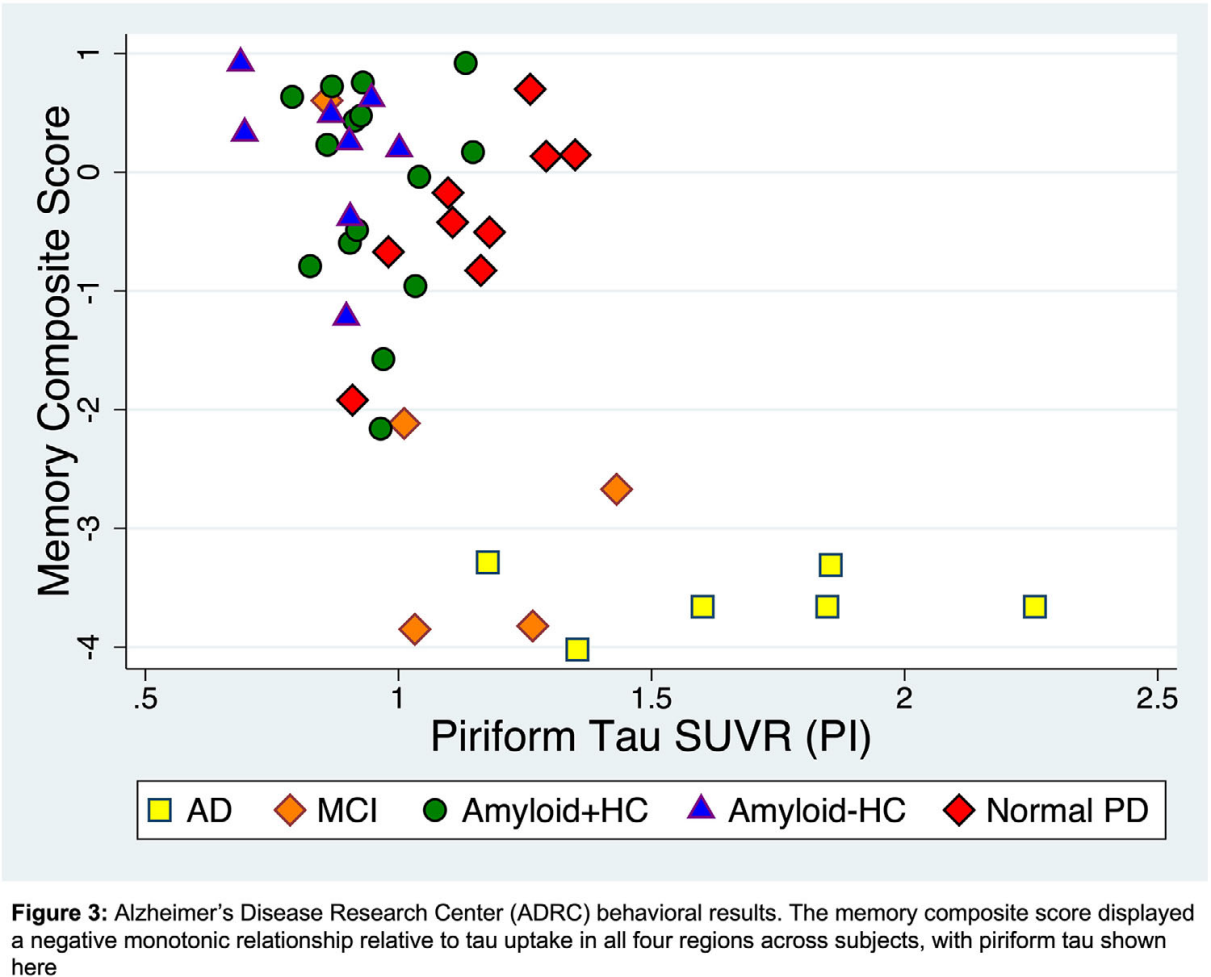# Elevated tau in the piriform cortex in Alzheimer’s but not Parkinson’s disease using MR‐PET

**DOI:** 10.1002/alz.092936

**Published:** 2025-01-03

**Authors:** Hossein Moein Taghavi, Mahta Karimpoor, Eric van Staalduinen, Samantha Leventis, Christina B. Young, Mackenzie L. Carlson, Guido A. Davidzon, America Romero, Alexandra N. Trelle, Greg Zaharchuk, Hillary Vossler, Jarrett Rosenberg, Kathleen L. Poston, Anthony D. Wagner, Victor W. Henderson, Marios Georgiadis, Elizabeth Mormino, Michael Zeineh

**Affiliations:** ^1^ Stanford University School of Medicine, Stanford, CA USA; ^2^ Wu Tsai Neuroscience Institute, Stanford, CA USA

## Abstract

**Background:**

Olfactory deficiency can be present in preclinical Alzheimer’s (AD) and Parkinson’s disease (PD), predicting their subsequent manifestation, including mild cognitive impairment (MCI). Analyzing key regions within the olfactory circuit could reveal important insights into the neuropathological progression. Dysfunction in the olfactory circuit has been shown in the olfactory nerve in limited postmortem studies, including involvement of a key region, the piriform cortex. FDG‐positron emission tomography (PET) and fMRI have shown differential and reduced piriform cortex metabolism/activation in AD. Thus, the piriform cortex is a promising candidate in the early identification of neurodegenerative pathology underlying olfaction. We used tau MR‐PET to analyze the piriform cortex, in comparison to the adjacent medial temporal lobe.

**Method:**

We analyzed 115 subjects: 23 were excluded (incomplete data), leaving 31 amyloid‐negative and 25 amyloid‐positive healthy controls, 8 MCI, 15 AD, and 13 PD. All subjects underwent MR‐PET using tau tracer PI‐2620 with a simultaneous coregistered sagittal T1‐weighted 3D IR‐FSPGR and a simultaneous/recently acquired coronal T2‐weighted FSE. Automatic Segmentation of Hippocampal Subfields was performed, including the entorhinal‐perirhinal cortices **(Fig. 1D‐F)**. Referencing published piriform segmentation guidelines, we manually segmented blind to subject diagnosis the frontal/temporal portions of the piriform cortex **(Fig. 1C)**, including multiple independent quality control checks. As tau distributions appeared non‐normal among the five ordinal patient categories (Amyloid–HC, Amyloid+HC, MCI, AD, Normal PD), we used a nonparametric Jonckheere‐Terpstra test in Stata to test for ordinal increase in tau uptake across four regions bilaterally (whole hippocampus = CA1‐4 + DG + subiculum, entorhinal‐perirhinal, amygdala, and piriform).

**Result:**

We found an ordinal increase in piriform tau uptake with increasing disease severity (amyloid‐negative controls, amyloid‐positive controls, MCI and AD) **(Figure 2A‐D)**. Amyloid‐positive controls showed significantly greater uptake than amyloid‐negative controls. Piriform uptake was not elevated in cognitively unimpaired PD compared to Amyloid‐HC. Piriform volume was statistically equivalent across groups, except PD had lower volumes **(Figure 2E‐H)**. Negative correlations were present between memory performance and piriform uptake **(Figure 3)**.

**Conclusion:**

Cross‐sectionally, there is an early increase in tau uptake in the piriform cortex in AD but not in PD.